# *n*-3 Fatty acid intake and circadian syndrome in US adults: evidence from the National Health and Nutrition Examination Survey 2005–2018

**DOI:** 10.1017/S0007114526106679

**Published:** 2026-06-28

**Authors:** Dan Zhang, Dandan Sun, Bizhong Che, Bin Zhang, Lei Zhao, Youfa Wang, Zumin Shi, Wen Peng

**Affiliations:** 1 School of Civil and Hydraulic Engineering, Qinghai University, Xining, Qinghai, 810016, China; 2 Department of Public Health, Qinghai University Medical College, Xining, Qinghai, 810008, China; 3 Nutrition and Health Promotion Center, Qinghai University Medical College, Xining, Qinghai, 810008, China; 4 Global Health Institute, School of Public Health, Xi’an Jiaotong University, Xi’an, Shaanxi, 710061, China; 5 School of Mathematics and Statistics, Qinghai Minzu University, Xining, Qinghai, 810007, China; 6 Department of Nutrition Sciences, College of Health Sciences, QU Health, Qatar University, Doha, Qatar; 7 Qinghai Provincial Key Laboratory of Prevention and Control of Glucolipid Metabolic Diseases with Traditional Chinese Medicine, Medical College, Qinghai University, Xining, Qinghai, 810008, China

**Keywords:** Circadian syndrome, Metabolic syndrome, *n*-3, National Health and Nutrition Examination Survey

## Abstract

Circadian syndrome (CircS) appears to be a better predictor for CVD than metabolic syndrome, and *n*-3 fatty acids are generally suggested to alleviate negative health outcomes. This study aims to investigate the association between *n*-3 intake and CircS and examine the effect modification by sociodemographic and lifestyle factors. In this cross-sectional study, data from the National Health and Nutrition Examination Survey 2005–2018 were analysed (*n* 12 028). Survey-weighted multivariable logistic regression was applied to analyse the associations of *n*-3 intake with CircS and its components. Subgroup analyses examining the effect modification by sociodemographic and lifestyle factors were followed by a restricted cubic spline investigating the non-linear associations between *n*-3 intake and CircS by race. The weighted prevalence of metabolic syndrome and CircS was 45·8 and 37·3 %, respectively. Overall, no significant associations were found between the intake of total *n*-3, EPA or DHA and CircS. However, *n*-3 intake was associated with a lower risk of depression – a component of CircS. Comparing extreme quartiles of *n*-3 intake (highest *v*. lowest), the OR (95 % CI) for depressive symptoms was 0·77 (0·64, 0·90). Among the Black, those in the highest quartile of *n*-3 intake were more likely to have CircS, with the OR (95 % CI) of 1·36 (1·02, 1·82). No association between *n*-3 intake and CircS in the total study population was observed. However, *n*-3 intake was inversely associated with depressive symptoms in American adults. Interactions between race and *n*-3 intake in relation to CircS were also identified.

Metabolic syndrome (MetS) has been identified as a cluster of physiological abnormalities leading to cardiovascular and other chronic diseases^(1)^. A recent study found that circadian dysfunction could be an important underlying aetiological factor for this clustering, and the concept of MetS has been expanded to circadian syndrome (CircS), incorporating additional co-morbidities such as disturbances in sleep patterns and depressive symptoms^(2)^. Moreover, this newly defined concept has been found to be a better predictor for CVD than MetS^(3)^.

Circadian regulation primarily depends on the alignment of the biological clock with synchronisers, which are external stimuli in the environment^(4)^. Representative synchronisers are daily light and dark cycles^(5)^, while certain nutrients such as caffeine and dietary polyphenols have been found to act as non-phonic synchronisers^(6,7)^. *n*-3 Fatty acids (FA) were also found to play a crucial role in circadian clock synchronisation as a non-phonic synchroniser^(4)^. Studies have demonstrated a negative association between DHA concentrations and melatonin levels in the pineal gland of rats^(8)^. Specifically, *n*-3 deficiency leads to reduced pineal melatonin secretion, which in turn decreases dopamine levels in the brain and disrupts sleep patterns^(9)^. *n*-3 FA have been shown to modulate various physiological processes, including inflammation, neurological functions, metabolism, reproductive functions, cardiovascular health and other biochemical pathways^(5)^through the regulation of clock genes. The core mechanism at the molecular level is gene expression operating based on transcriptional/translational feedback loops^(10)^, in which two transcription factors – circadian locomotor output cycles kaput and brain and muscle Arnt-like protein 1 – were involved.

Extensive studies have explored the association between *n*-3 and MetS^(11)^. It was found that dietary *n*-3 intake increased hepatic AMPK activity *in vivo*, promoting FA oxidation and reducing adiposity^(12)^. Dietary DHA and EPA modulate co-cultured human macrophages and adipocytes by suppressing pro-inflammatory cytokine expression and lowering serum TAG. Upon consumption, *n*-3 FA undergo competitive metabolic processing involving desaturation, elongation and *β*-oxidation pathways. This metabolism generates anti-inflammatory lipid mediators that counteract insulin resistance and obesity-related hepatic steatosis^(13)^. Intake of *n*-3 was reported to be inversely associated with glucose levels and the prevalence of diabetes^(14)^. *n*-3 FA were reported to be was reported beneficial in controlling blood pressure^(15)^ and HDL-cholesterol^(16)^.

The effects of *n*-3 on sleep disturbances were also investigated. A recent review highlighted the effects of *n*-3 on sleep quality and circadian processes; *n*-3 and DHA in particular were found promising for improving sleep quality and limiting the adverse effects of sleep disturbance on health^(17)^. Another study indicated that *n*-3 intake was correlated with very short sleep duration, and these associations were contingent upon sex and age^(18)^. Furthermore, negative associations between *n*-3 and depression were discovered, regardless of whether it was the female group^(19)^ or the older adult group^(20)^.

Despite these findings, no previous studies have investigated the association between *n*-3 and CircS. Using data from the National Health and Nutrition Examination Survey (NHANES), this study examined the association between dietary *n*-3 FA intake and CircS and investigated how sociodemographic and lifestyle factors may modify these associations.

## Methods and materials

### Study design and sample

**Figure 1.**
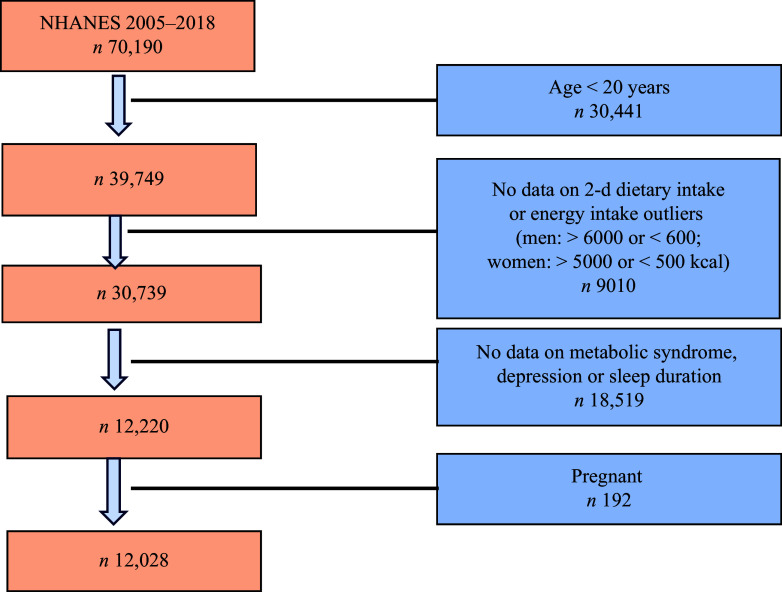
Study sample flow chart: National Health and Nutrition Examination Survey (NHANES) data used in the analysis.

This cross-sectional study utilised data from the National Health and Nutrition Examination Survey (NHANES) spanning 2005–2018. Of the 39 749 eligible participants aged ≥ 20 years, we further excluded those with missing 2-d dietary intake data or implausible energy intake (*n* 9010), followed by exclusion of participants lacking data on MetS, depression or sleep duration (*n* 18 519). Pregnant women (*n* 192) were also excluded^(21)^, resulting in a final analytical sample of 12 028 adults (Figure 1).

### Outcome variables

The primary outcome measure of this study was CircS. The definition of this syndrome was based on the core components proposed by Zimmet *et al.* ^(22)^, and its operational definition was proposed by Shi *et al*.^(3)^ and used in a population study among Chinese adults. A participant was classified as having CircS if they met four or more of the following seven components. The first five components are consistent with the diagnostic criteria for MetS^(23)^, and their specific diagnostic thresholds and scoring methods are detailed below:

1. Elevated waist circumference: ≥ 102 cm for men and ≥ 88 cm for women.
2. Elevated blood pressure: Systolic blood pressure ≥ 130 mm Hg and/or diastolic blood pressure ≥ 85 mm Hg.
3. Reduced HDL-cholesterol: < 40 mg/dl (1·04 mmol/l) for men and < 50 mg/dl (1·30 mmol/l) for women, or receiving drug treatment for reduced HDL-cholesterol.
4. Elevated TAG: ≥ 150 mg/dl (1·70 mmol/l), or receiving drug treatment for dyslipidemia.
5. Elevated fasting glucose: ≥ 100 mg/dl (5·6 mmol/l), or receiving drug treatment for elevated glucose.
6. Short sleep duration: Self-reported average sleep duration < 6 h/d.
7. Depressive symptoms: A score of ≥ 5 on the Patient Health Questionnaire-9.

Compared with MetS (diagnosed when three or more of the five components are met), CircS incorporates two new components and raises the diagnostic threshold. While retaining the core focus on assessing metabolic abnormalities, it more comprehensively captures the impacts of circadian rhythm and psychological status on health. The optimisation of this diagnostic criterion enables the identification of individuals with isolated CircS who do not meet MetS criteria, highlighting the added value of integrating sleep and mental health-related components into its definition^(22)^.

### Exposure variables

Dietary intake was assessed using two non-consecutive 24-h dietary recall interviews, a widely used method for assessing population-level dietary patterns in large surveys^(24)^. The first interview was conducted in person at the Mobile Examination Center, and the second was conducted by telephone 3–10 d later to account for day-to-day variation in intake. The mean daily intake (mg/d) of *n*-3 FA from food sources was calculated by averaging the amounts reported across the two recalls. These calculations were performed using the USDA’s Food and Nutrient Database for Dietary Studies^(25,26)^, which provides the nutrient composition for each food and beverage reported. Total energy intake (kcal/d) was calculated in the same manner by summing the energy contributions from all consumed foods and beverages. Participants with missing 2-d dietary recall data were excluded. To minimise the influence of implausible energy intake reporting on the results, we further excluded individuals with extreme daily energy intake, using the widely adopted criteria of ^(21,25)^< 600 kcal or > 6000 kcal for men and < 500 kcal or > 5000 kcal for women (*n* 9010). This approach is standard in NHANES analyses to exclude physiologically implausible data that likely result from misreporting, acute dietary changes or underlying health conditions, thereby reducing potential bias in estimating diet-disease associations^(23,27)^. Furthermore, in a sensitivity analysis, we applied a more conservative cut-off for energy intake (3500 kcal/d for women and 4200 kcal/d for men)^(28)^ and found that the primary results remained unchanged, confirming the robustness of our findings.

In this study, we focused specifically on long-chain *n*-3 FA, namely, EPA and DHA, due to their well-established biological activities and strong evidence base regarding their potential benefits for metabolic and circadian health^(29,30)^. The primary dietary sources of EPA and DHA in this cohort were fish and seafood products (e.g. salmon, tuna, shrimp)^(27)^, which is consistent with the established understanding of the US diet and findings from NHANES. Other *n*-3 FA (e.g. *α*-linolenic acid) or *n*-6 FA were not the primary focus of this investigation, as their mechanisms and associations with the outcome may differ. Similarly, while other nutrients like dietary fibre and antioxidants may influence health outcomes, the present analysis specifically aimed to examine the independent role of pre-specified *n*-3 FA (EPA and DHA).

Our assessment of *n*-3 FA exposure was limited to intake from dietary sources alone, as estimated from the 24-h dietary recalls. We did not integrate intake from dietary supplements into our primary exposure variable due to inconsistencies in the supplemental nutrient data across NHANES cycles. As such, the potential confounding effects of overall diet quality were addressed by adjusting for the Healthy Eating Index (HEI) in sensitivity analyses (model 3).

### Covariates

Covariates included in this study were selected based on evidence from nutritional epidemiology and circadian rhythm research, as well as established confounding pathways linking each variable to both the exposure (*n*-3 intake) and the outcome (CircS). Specifically, three categories of factors potentially influencing the results were accounted for: demographic characteristics, behavioural factors and health-related variables.

Demographic characteristics include age, sex, race (subdivided into non-Hispanic (NH) White, non-Hispanic, Mexican American and other races), education (under high school, high school, some college, college and above) and the ratio of household income:poverty line (< 1·30, 1·3–3·5 and > 3·5). These variables are all standard adjustment factors in NHANES-based nutritional epidemiology studies^(31–33)^, and previous research has validated that they serve as well-recognised fundamental confounders in this field^(34–36)^.

Alcohol consumption (assessed by self-reporting alcohol consumption over the past 12 months, divided into three categories: yes, no and missing), smoking status (never, former and current smoker) and leisure-time physical activity (categorised into three levels based on the physical activity guidelines from the American Heart Association and the American College of Sports Medicine^(37)^: < 600 Metabolic Equivalent of Task (MET)-min/week, 600–1200 MET-min/week, > 1200 MET-min/week) were included as behavioural factors. These cut-offs correspond to activity levels below the recommended level, meeting the minimum recommendation (equivalent to ≥ 150 min of moderate-intensity activity) and exceeding twice the minimum recommendation, respectively^(38)^. Health-related variables included the HEI and dietary supplement use. The HEI-2015 score, a measure of overall diet quality that assesses compliance with the US Dietary Guidelines for Americans, was calculated from the two 24-h dietary recalls^(39)^. The calculation was performed using the simple HEI scoring algorithm developed by the National Cancer Institute implemented in SAS code by researchers at Emory University. Total scores range from 0 to 100, with higher scores indicating better diet quality. Dietary supplement use (yes/no) was defined as the reported use of any vitamins, minerals or other nutritional supplements in the past 30 d, as obtained from the household interview^(40)^. Similarly, the inclusion of these variables as covariates in this study is justified by the fact that they have been validated in previous studies to simultaneously influence *n*-3 intake and CircS risk and are recognised as key confounders in nutritional epidemiology^(41–46)^.

### Statistical analysis

Following the NHANES analytical guidelines, we used a 2-year fasting subsample Mobile Examination Center weight (wtsf2yr) to account for the survey design of NHANES data in all analyses^(47)^. The combined sampling weight for the seven cycles (wtsf14yr) was calculated by dividing wtsf2yr by 7. The strata variable was sdmvstra, and the cluster (primary sampling unit) was sdmvpsu. The following Stata command was used to set the survey design: svyset [w = wtsf14yr], psu(sdmvpsu) strata(sdmvstra) singleunit(centered).

Participant characteristics were presented as weighted means and sd for continuous measures and unweighted frequency (weighted %) for categorical measures, respectively, across the quartiles of *n*-3 intake. Linear regression and the *χ* ^2^ test were used to test the group difference of continuous variables and categorical variables, respectively.

Survey-weighted multivariable logistic regression was used to analyse the association of *n*-3, EPA and DHA with CircS. Model 1 was adjusted for age, gender and energy intake. Model 2 was further adjusted for race, physical activity, education level, income, smoking and alcohol consumption. Model 3 was further adjusted for the HEI (by integrating dietary fibre, antioxidants and other nutrients into a holistic measure of dietary quality) and provided correlations across different models (expressed as a ratio and 95 % CI). Model 4 was additionally adjusted for dietary supplement use.

A total of eight variables (education, smoking, HEI, dietary supplement use, BMI, physical activity and income) had missing data in the full multivariable model. In accordance with the three-dimensional principle proposed by Dong and Peng^(48)^, missing values for alcohol drinking were categorised as a separate group (rather than excluded) for analysis, given its high percentage of missing(23·8 %, exceeding the 20 % threshold). For the remaining seven variables, complete case analysis was employed to exclude observations with missing values. This strategy is justified by their consistently low missing rates(< 5 %) – a threshold demonstrated to exert no material impact on statistical inference^(49)^.

Model 2 was considered our primary model to estimate the total association between *n*-3 intake and CircS. Models 3 and 4, which included additional adjustments for the HEI and dietary supplement use, were conducted as sensitivity analyses to examine the robustness of the findings, validating the robustness of our primary findings.

Subgroup analyses were conducted to examine the interactions between types of *n*-3 intake and factors such as age, sex, race, education, smoking status, alcohol consumption, leisure-time physical activity and household income:poverty line ratio. A restricted cubic spline method was applied to further explore the non-linear association between *n*-3 intake and CircS across races (White, Black, Mexican American, etc.), in which three knots were set at the 10th, 50th and 90th percentiles.

All statistical analyses were performed using Stata software, version 18·5 (StataCorp LLC).

## Results

### Characteristics of participants

Table 1 shows the characteristics of the study population by quartiles of *n*-3 intake. The weighted mean age (sd) of participants was 48·0 (15·1) years. Female and male participants accounted for 51·4 and 48·6 %, respectively, and 70·0 % of the study population was NH White. The weighted prevalences of MetS and CircS were 45·8 and 37·3 %, respectively. The total mean intake of *n*-3 FA was 109·6 mg/d, with mean intakes across quartiles Q1 to Q4 being 7·2 mg/d, 24·5 mg/d, 57·9 mg/d and 366·0 mg/d, respectively. This intake exhibited a significant, gradual increasing trend from Q1 to Q4 (*P* < 0·001).

**Table 1.** Baseline characteristics of the study population, stratified by quartiles of *n*-3 fatty acid intake: National Health and Nutrition Examination Survey 2005–2018 Table 1 long description.

	Quartiles of *n*-3 FA										
	Q1		Q2		Q3		Q4		Total		*P*
	Mean	sd	Mean	sd	Mean	sd	Mean	sd	Mean	sd	
*n*	3046	26·2	2992	25·8	2987	24·2	3003	23·9	12 028	100·0	
Age (years)	48·0	15·4	47·7	15·2	47·5	15·0	48·6	14·6	48·0	15·1	0·284
Sex											
Men	1314	41·6	1393	48·4	1583	52·3	1598	52·8	5888	48·6	< 0·001
Women	1732	58·4	1599	51·6	1404	47·7	1405	47·2	6140	51·4	
Race											
NH White	1588	74·4	1483	72·9	1314	68·5	1149	63·5	5534	70·0	< 0·001
NH Black	470	8·2	522	8·9	638	11·6	722	12·9	2352	10·3	
Mexican American	391	6·4	453	7·5	515	9·1	447	8·2	1806	7·8	
Other race/ethnic	597	10·9	534	10·7	520	10·8	685	15·4	2336	11·9	
Education											
< High school	761	16·5	657	14·1	713	14·6	625	12·8	2756	14·6	< 0·001
High school	731	24·6	728	25·0	677	21·9	623	20·9	2759	23·2	
Some college	902	32·1	914	31·0	886	31·9	886	31·0	3588	31·5	
College and above	650	26·8	690	29·8	709	31·6	868	35·3	2917	30·8	
Alcohol drinking (past 12 months)											
No	512	13·5	514	13·9	479	12·2	433	11·9	1938	12·9	0·003
Yes	1679	59·7	1773	62·6	1788	65·5	1903	65·8	7143	63·3	
Missing	855	26·8	705	23·4	720	22·2	667	22·3	2947	23·8	
Smoking											
Never	1622	53·3	1613	54·6	1619	54·6	1700	55·9	6554	54·6	0·014
Former	761	24·8	768	25·4	784	27·1	781	27·7	3094	26·2	
Current smoker	663	21·9	609	20·0	584	18·2	520	16·4	2376	19·2	
Energy intake (kcal/d)	1818·5	615·3	2085·0	662·8	2238·7	712·8	2272·4	770·2	2097·1	707·7	< 0·001
*n*-3 FA intake (mg/d)	7·2	3·8	24·5	5·7	57·9	14·3	366·0	383·2	109·6	224·6	< 0·001
EPA intake (mg/d)	3·6	2·6	7·8	4·4	13·1	7·7	127·8	154·5	36·6	87·1	< 0·001
DHA intake (mg/d)	3·6	2·6	7·8	4·4	13·1	7·7	127·8	154·5	36·6	87·1	< 0·001
Healthy Eating Index	44·3	11·8	44·8	10·9	45·6	10·9	49·5	11·0	46·0	11·3	< 0·001
Dietary supplement use	1549	53·2	1554	53·7	1550	53·7	1715	60·3	6368	55·2	< 0·001
BMI (kg/m^2^)	28·7	5·8	29·0	5·9	29·3	6·2	29·1	6·4	29·0	6·1	0·036
Leisure-time physical activity											
< 600 MET-min/week	1309	38·5	1170	34·2	1162	33·7	1059	31·3	4700	34·5	< 0·001
600–1200 MET-min/week	311	10·3	364	12·8	339	11·8	349	12·5	1363	11·8	
> 1200 MET-min/week	1425	51·2	1458	53·0	1486	54·5	1595	56·3	5964	53·7	
Ratio of family income:poverty											
< 1·30	957	24·0	866	20·8	784	17·8	660	15·7	3267	19·7	< 0·001
1·3–3·5	1073	36·8	1099	39·0	1082	37·1	1060	34·9	4314	37·0	
> 3·5	785	39·2	803	40·3	886	45·1	10 309	49·4	3504	43·3	
Central obesity	1795	59·3	1771	57·2	1768	58·4	1658	55·6	6992	57·7	0·128
Elevated glucose	1639	49·5	1599	50·8	1658	52·4	1695	53·0	6591	51·4	0·190
Elevated TAG	1338	41·5	1251	38·9	1261	39·4	1278	40·7	5128	40·1	0·368
Reduced HDL-cholesterol	1448	44·8	1330	41·5	1402	44·0	1322	42·3	5502	43·2	0·262
Elevated blood pressure	1505	42·9	1455	43·4	1508	45·6	1473	43·0	5941	43·7	0·336
Depression	807	25·3	756	23·2	625	18·4	629	19·0	2817	21·6	< 0·001
Short sleep	1000	30·2	977	29·3	989	28·7	1003	29·2	3969	29·4	0·790
Metabolic syndrome	1551	46·6	1438	43·9	1521	47·2	1463	45·8	5973	45·8	0·248
Circadian syndrome	1314	38·5	1216	35·5	1269	37·7	1237	37·3	5036	37·3	0·273

FA, fatty acid; NH, non-Hispanic.

Data were presented as weighted means and standard deviations for continuous measures and unweighted frequency (weighted %) for categorical measures, respectively. The *P*-value is weighted. *P*-values are based on linear regression for continuous measures and survey-weighted multivariable logistic regression for categorical measures.

Number of missing: education (8), smoking (4), dietary supplement use (6), BMI (58), physical activity (1), income (943).

Across *n*-3 FA intake quartiles, notable differences emerged in both sex and racial/ethnic distributions. Specifically, male participants and individuals identifying as NH Black were progressively overrepresented in higher quartiles of *n*-3 FA intake (both *P*_for trend_ < 0·001). Participants with high *n*-3 intake (Q4) were more likely to be engaged in alcohol drinking, be non-smokers, participate in heavy leisure-time physical activity, have higher education levels and come from families with good economic conditions. In addition, energy intake, EPA and DHA intake, BMI and depression all varied with *n*-3 intake.

### Associations between *n*-3 intake quartiles and circadian syndrome and its components

Table 2 shows the association between *n*-3 intake and CircS. Intake of *n*-3, EPA and DHA was not associated with CircS, after adjusting for age, race, physical activity, education, income, smoking, alcohol drinking, HEI and dietary supplement use.

**Table 2.** Association between dietary *n*-3 fatty acid (FA) intake and circadian syndrome among US adults: National Health and Nutrition Examination Survey 2005–2018 Table 2 long description.

Quartiles of *n*-3 FA								
		Q2		Q3		Q4		*P*_for trend_
	Q1	OR	95 % CI	OR	95 % CI	OR	95 % CI	
*n*-3 FA								
Model 1	1·00	0·86	0·74, 0·99	0·96	0·83, 1·10	0·88	0·75, 1·04	0·300
Model 2	1·00	0·88	0·76, 1·02	1·02	0·88, 1·18	0·98	0·83, 1·15	0·779
Model 3	1·00	0·89	0·76, 1·03	1·04	0·90, 1·20	1·03	0·87, 1·22	0·360
Model 4	1·00	0·89	0·76, 1·03	1·03	0·90, 1·19	1·03	0·87, 1·22	0·359
EPA								
Model 1	1·00	1·00	0·84, 1·20	1·03	0·88, 1·21	0·89	0·76, 1·06	0·221
Model 2	1·00	1·02	0·86, 1·21	1·06	0·90, 1·26	0·99	0·83, 1·17	0·974
Model 3	1·00	1·01	0·85, 1·21	1·05	0·89, 1·25	1·02	0·86, 1·21	0·703
Model 4	1·00	1·01	0·85, 1·20	1·06	0·89, 1·25	1·02	0·86, 1·21	0·668
DHA								
Model 1	1·00	0·84	0·72, 0·98	0·92	0·79, 1·06	0·87	0·74, 1·03	0·180
Model 2	1·00	0·88	0·76, 1·03	0·98	0·84, 1·13	0·96	0·82, 1·14	0·918
Model 3	1·00	0·89	0·77, 1·04	1·00	0·86, 1·16	1·02	0·86, 1·20	0·573
Model 4	1·00	0·89	0·76, 1·04	0·99	0·86, 1·15	1·02	0·86, 1·21	0·576

Model 1 adjusted for age, gender and energy intake; model 2 further adjusted for race, physical activity, education, income, smoking and alcohol drinking; model 3 further adjusted for Healthy Eating Index; model 4 further adjusted for dietary supplement use.

However, a significant relationship was observed when the CircS was stratified into seven components in model 2 (Table 3). An increase of *n*-3 intake was associated with decreasing depression risk, with the OR (95 % CI) of upper quartiles of *n*-3 intake being 0·71 (0·60, 0·83) and 0·77 (0·64, 0·91), respectively (*P*_for trend_ < 0·001). Both high EPA and DHA intake were associated with a lower risk of depressive symptoms; the OR (95 % CI) of upper quartiles of EPA intake were 0·82 (0·70, 0·96) and 0·75 (0·63, 0·89), respectively, and the OR (95 % CI) of upper quartiles of DHA intake were 0·71 (0·60, 0·83) and 0·77 (0·64, 0·91), respectively (both *P*_for trend_ ≤ 0·001).

**Table 3.** Weighted OR (95 % CI) for components of circadian syndrome across quartiles of *n*-3 fatty acid (FA) intake: National Health and Nutrition Examination Survey 2005–2018 Table 3 long description.

Quartiles of intake								
		Q2		Q3		Q4		*P*_for trend_
	Q1	OR	95 % CI	OR	95 % CI	OR	95 % CI	
*n*-3 FA								
Central obesity	1·00	0·99	0·86, 1·13	1·09	0·95, 1·24	0·97	0·85, 1·12	0·948
Elevated glucose	1·00	1·04	0·89, 1·23	1·11	0·93, 1·31	1·10	0·92, 1·31	0·227
Elevated TAG	1·00	0·89	0·76, 1·04	0·93	0·81, 1·08	0·97	0·84, 1·12	0·827
Low HDL-cholesterol	1·00	0·91	0·77, 1·06	1·05	0·93, 1·19	0·97	0·83, 1·13	0·801
Elevated blood pressure	1·00	1·04	0·89, 1·22	1·18	1·00, 1·40	0·95	0·80, 1·13	0·928
Depressive symptom	1·00	0·93	0·80, 1·08	0·70	0·60, 0·82	0·76	0·64, 0·90	< 0·001
Short sleep	1·00	0·93	0·81, 1·07	0·88	0·76, 1·02	0·90	0·78, 1·05	0·135
EPA								
Central obesity	1·00	1·01	0·89, 1·15	1·12	0·98, 1·27	1·01	0·87, 1·18	0·576
Elevated glucose								
Elevated TAG	1·00	1·02	0·87, 1·18	0·96	0·81, 1·13	0·95	0·83, 1·10	0·384
Low HDL-cholesterol	1·00	1·05	0·91, 1·22	1·11	0·95, 1·30	1·01	0·87, 1·16	0·759
Elevated blood pressure	1·00	1·02	0·86, 1·20	1·03	0·88, 1·20	0·97	0·81, 1·15	0·731
Depressive symptom	1·00	0·88	0·75, 1·04	0·82	0·70, 0·96	0·75	0·63, 0·89	0·001
Short sleep	1·00	0·90	0·78, 1·05	0·98	0·84, 1·14	0·90	0·77, 1·05	0·299
DHA								
Central obesity	1·00	1·04	0·91, 1·19	1·09	0·94, 1·25	0·93	0·81, 1·08	0·523
Elevated glucose	1·00	0·96	0·82, 1·14	1·01	0·85, 1·20	1·02	0·87, 1·21	0·671
Elevated TAG	1·00	0·92	0·79, 1·06	0·91	0·78, 1·05	0·96	0·83, 1·11	0·539
Low HDL-cholesterol	1·00	0·98	0·85, 1·13	1·02	0·89, 1·17	0·99	0·85, 1·16	0·930
Elevated blood pressure	1·00	1·08	0·91, 1·27	1·14	0·95, 1·38	0·95	0·79, 1·14	0·784
Depressive symptom	1·00	0·85	0·72, 1·01	0·71	0·60, 0·83	0·77	0·64, 0·91	< 0·001
Short sleep	1·00	0·98	0·85, 1·13	0·91	0·79, 1·06	0·92	0·79, 1·08	0·224

Model adjusted for age, gender, energy intake, race, physical activity, education, income, smoking and alcohol drinking.

Subsequent to identifying the association between *n*-3 intake and CircS in the primary model (model 2), sensitivity analyses (models 3 and 4) were performed to assess the robustness of this primary finding. Model 3 incorporated additional adjustments for the HEI, while model 4 extended these adjustments by further accounting for dietary supplement use, building upon the covariates included in model 3. Sensitivity analyses revealed that after accounting for these additional variables, no statistically significant alterations were noted in either the magnitude or direction of the weighted OR characterising the association between *n*-3 intake and CircS (online Supplementary Table S2).

### Subgroup analysis

**Figure 2.**
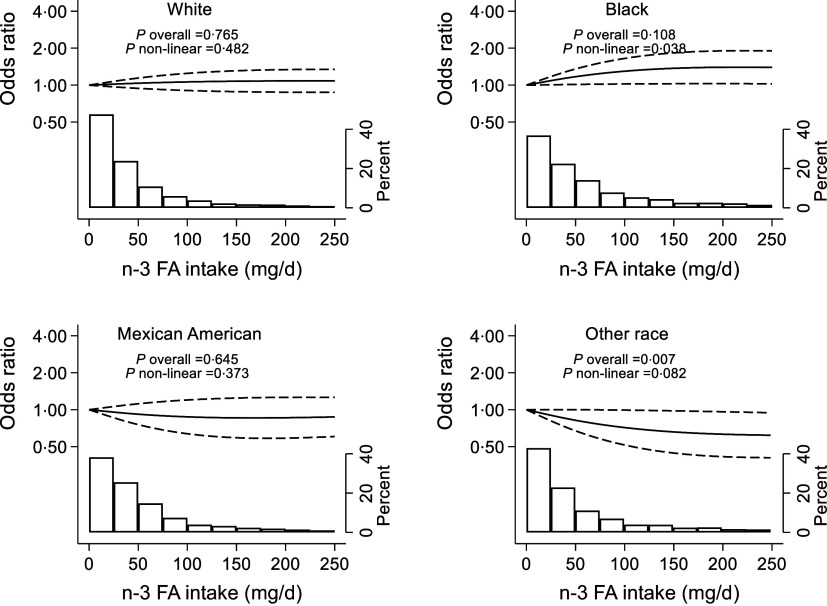
Non-linear association between *n*-3 fatty acid (FA) intake and circadian syndrome by race among adults attending the National Health and Nutrition Examination Survey 2005–2018. Variables adjusted were the same as model 2 in Table 2. The restricted cubic spline method was used with knots put at the 10th, 50th and 90th percentiles. The solid line represents OR, and the dashed lines represent 95 % CI of the OR. The histogram shows the distribution of *n*-3 FA intake.

Subgroup analysis was conducted based on the final model (model 2) to test the modification effect of sociodemographic and lifestyle factors on the associations between *n*-3 intake and CircS. As shown in Table 4, interactions between race and *n*-3 intake in relation to CircS were identified. Among participants from the NH Black group, those in the highest quartile of *n*-3 intake were more likely to have CircS compared with those in the lowest quartile, with an OR (95 % CI) of 1·36 (1·02, 1·82). Furthermore, the association between *n*-3 intake and CircS among participants from the NH Black was significantly non-linear (*P*_non-linear_ = 0·038), and the high *n*-3 intake was associated with an increasing CircS risk (Figure 2).

**Table 4.** Subgroup analysis of the association between quartiles of *n*-3 fatty acid intake and circadian syndrome: National Health and Nutrition Examination Survey 2005–2018 Table 4 long description.

	Quartiles of *n*-3 FA								
		Q2		Q3		Q4		*P*_for trend_	*P*_for interaction_
	Q1	OR	95 % CI	OR	95 % CI	OR	95 % CI		
Age									0·317
20–39	1·00	0·94	0·69, 1·28	0·95	0·69, 1·33	0·67	0·48, 0·95	0·054	
40–59	1·00	0·86	0·67, 1·11	1·00	0·78, 1·28	1·03	0·78, 1·37	0·603	
60+	1·00	0·85	0·64, 1·14	1·03	0·80, 1·32	1·05	0·83, 1·33	0·342	
Sex									0·064
Men	1·00	0·87	0·72, 1·04	1·15	0·95, 1·39	1·08	0·87, 1·33	0·125	
Women	1·00	0·91	0·72, 1·14	0·93	0·75, 1·16	0·92	0·74, 1·16	0·522	
Race									0·008
NH White	1·00	0·87	0·72, 1·06	1·07	0·89, 1·30	1·05	0·85, 1·30	0·310	
NH Black	1·00	1·10	0·81, 1·50	1·16	0·87, 1·54	1·36	1·02, 1·82	0·032	
Mexican American	1·00	0·78	0·53, 1·14	0·87	0·59, 1·29	0·83	0·60, 1·14	0·401	
Other race/ethnic	1·00	0·85	0·59, 1·23	0·78	0·52, 1·17	0·57	0·39, 0·85	0·006	
Education									0·105
< High school	1·00	1·17	0·86, 1·58	1·22	0·90, 1·64	1·11	0·80, 1·55	0·427	
High school	1·00	0·84	0·63, 1·12	1·16	0·84, 1·61	1·38	0·93, 2·04	0·037	
Some college	1·00	0·78	0·61, 1·01	0·89	0·69, 1·16	0·79	0·60, 1·04	0·190	
College and above	1·00	0·96	0·65, 1·43	1·07	0·77, 1·47	1·00	0·72, 1·38	0·835	
Smoking									0·653
Never	1·00	0·97	0·78, 1·22	1·16	0·96, 1·41	1·04	0·85, 1·28	0·330	
Former	1·00	0·71	0·54, 0·94	0·86	0·65, 1·14	0·87	0·65, 1·17	0·654	
Current smoker	1·00	0·97	0·72, 1·31	0·96	0·68, 1·36	1·00	0·67, 1·51	0·985	
Alcohol drinking (past 12 months)									0·771
No	1·00	0·91	0·63, 1·31	1·00	0·70, 1·43	1·18	0·83, 1·68	0·310	
Yes	1·00	0·88	0·71, 1·08	1·08	0·89, 1·32	0·96	0·76, 1·21	0·810	
Missing	1·00	0·90	0·65, 1·23	0·87	0·61, 1·24	0·94	0·67, 1·30	0·662	
Leisure-time physical activity									0·572
< 600 MET-min/week	1·00	0·89	0·69, 1·14	1·20	0·94, 1·52	1·05	0·81, 1·36	0·272	
600–1200 MET-min/week	1·00	1·34	0·86, 2·07	1·10	0·68, 1·80	0·98	0·61, 1·58	0·672	
> 1200 MET-min/week	1·00	0·79	0·64, 0·98	0·89	0·71, 1·13	0·95	0·75, 1·20	0·947	
Ratio of family income:poverty									0·078
< 1·30	1·00	0·82	0·63, 1·05	0·99	0·73, 1·33	1·05	0·80, 1·37	0·575	
1·3–3·5	1·00	0·89	0·68, 1·17	1·02	0·81, 1·30	0·90	0·70, 1·15	0·665	
> 3·5	1·00	0·92	0·68, 1·25	1·17	0·92, 1·50	1·10	0·83, 1·45	0·232	
Dietary supplement use									0·464
No	1·00	0·86	0·71, 1·04	1·02	0·84, 1·23	1·07	0·86, 1·33	0·303	
Yes	1·00	0·89	0·70, 1·12	1·01	0·81, 1·25	0·91	0·73, 1·14	0·671	

FA, fatty acid; NH, non-Hispanic.

Model adjusted for race, physical activity, education, income, smoking and alcohol drinking.

The curve experiences a growth phase at the outset, during which the rate of increase is high. However, as the *n*-3 intake increases continuously, the growth rate decelerates gradually, and the curve flattens out over time. Increasing *n*-3 intake was associated with a lower risk of CircS for participants from other races (Table 4), and Q4 had an OR of 0·57 (95 % CI 0·39, 0·85). The result of RCS also shows that high *n*-3 intake was associated with decreasing CircS risks (Figure 2), while the association between them is more likely to be linear (*P*_non-linear_ = 0·082).

Table 5 shows the association between the quartiles of EPA intake and CircS. There was a significant interaction between family income and EPA intake in relation to CircS. Among those with better family economic conditions, the association between the highest quartile of EPA intake and CircS was not statistically significant, although a positive trend was suggested (OR 1·27; 95 % CI 0·95, 1·68). Similarly, an interaction between race, education and DHA intake was found (Table 6). For participants from other races, DHA intake was associated with a lower risk of CircS. Moreover, among participants with a high school education background, those in the highest quartile of DHA intake were more likely to be diagnosed with CircS compared with those in the lower quartiles, with the OR (95 % CI) of 1·54 (1·08, 2·20).

**Table 5.** Subgroup analysis of the association between quartiles of EPA intake and circadian syndrome: National Health and Nutrition Examination Survey 2005–2018 Table 5 long description.

	Quartiles of EPA								
	Q1	Q2		Q3		Q4		*P*_for trend_	*P*_for interaction_
		OR	95 % CI	OR	95 % CI	OR	95 % CI		
Age									0·395
20–39	1·00	1·01	0·72, 1·42	0·95	0·70, 1·29	0·77	0·54, 1·09	0·127	
40–59	1·00	1·05	0·79, 1·40	1·21	0·94, 1·56	1·07	0·81, 1·41	0·438	
60+	1·00	0·96	0·76, 1·22	0·93	0·67, 1·28	0·99	0·78, 1·26	0·892	
Sex									0·063
Men	1·00	1·11	0·85, 1·44	1·12	0·89, 1·40	1·13	0·90, 1·43	0·294	
Women	1·00	0·95	0·77, 1·16	1·04	0·82, 1·32	0·89	0·71, 1·11	0·463	
Race									0·180
NH White	1·00	1·03	0·83, 1·29	1·07	0·86, 1·33	1·03	0·83, 1·29	0·689	
NH Black	1·00	0·80	0·57, 1·13	1·07	0·82, 1·41	1·18	0·88, 1·59	0·091	
Mexican American	1·00	0·83	0·60, 1·14	0·89	0·66, 1·21	0·79	0·60, 1·06	0·192	
Other race/ethnic	1·00	1·25	0·87, 1·79	1·11	0·72, 1·73	0·77	0·53, 1·11	0·106	
Education									0·562
< High school	1·00	1·12	0·85, 1·48	1·09	0·80, 1·49	1·06	0·79, 1·42	0·688	
High school	1·00	1·10	0·76, 1·58	1·09	0·78, 1·53	1·25	0·83, 1·88	0·299	
Some college	1·00	1·13	0·83, 1·53	1·06	0·82, 1·38	0·88	0·67, 1·14	0·262	
College and above	1·00	0·72	0·51, 1·02	1·02	0·71, 1·48	0·95	0·71, 1·29	0·650	
Smoking									0·776
Never	1·00	1·00	0·79, 1·26	1·14	0·90, 1·44	1·02	0·83, 1·25	0·612	
Former	1·00	0·95	0·72, 1·26	0·87	0·68, 1·12	0·97	0·72, 1·29	0·681	
Current smoker	1·00	1·13	0·80, 1·59	1·20	0·83, 1·74	0·91	0·59, 1·40	0·846	
Alcohol drinking (past 12 months)									0·050
No	1·00	0·77	0·58, 1·04	0·98	0·69, 1·39	0·90	0·65, 1·26	0·787	
Yes	1·00	1·07	0·83, 1·38	1·18	0·95, 1·47	1·10	0·88, 1·37	0·277	
Missing	1·00	1·04	0·69, 1·58	0·87	0·60, 1·28	0·79	0·54, 1·17	0·133	
Leisure-time physical activity									0·922
< 600 MET-min/week	1·00	0·94	0·75, 1·19	1·08	0·85, 1·36	0·97	0·76, 1·25	0·883	
600–1200 MET-min/week	1·00	1·18	0·75, 1·84	1·33	0·89, 2·00	0·89	0·56, 1·40	0·726	
> 1200 MET-min/week	1·00	1·03	0·80, 1·33	1·00	0·78, 1·29	1·03	0·82, 1·30	0·858	
Ratio of family income:poverty									0·004
< 1·30	1·00	0·90	0·68, 1·20	0·96	0·73, 1·27	1·02	0·78, 1·35	0·883	
1·3–3·5	1·00	1·02	0·79, 1·31	1·08	0·81, 1·44	0·84	0·64, 1·09	0·279	
> 3·5	1·00	1·20	0·86, 1·67	1·29	0·94, 1·76	1·27	0·95, 1·68	0·094	
Dietary supplement use									0·737
No	1·00	0·99	0·78, 1·25	1·08	0·85, 1·37	1·05	0·82, 1·34	0·544	
Yes	1·00	1·04	0·82, 1·31	1·06	0·85, 1·32	0·95	0·77, 1·17	0·632	

NH, non-Hispanic.

Model adjusted for race, physical activity, education, income, smoking and alcohol drinking.

**Table 6.** Subgroup analysis of the association between quartiles of DHA intake and circadian syndrome: National Health and Nutrition Examination Survey 2005–2018 Table 6 long description.

	Quartiles of DHA								
	Q1	Q2		Q3		Q4		*P*_for trend_	*P*_for interaction_
		OR	95 % CI	OR	95 % CI	OR	95 % CI		
Age									0·192
20–39	1·00	0·88	0·65, 1·19	0·88	0·62, 1·24	0·63	0·44, 0·90	0·023	
40–59	1·00	0·93	0·72, 1·20	1·01	0·81, 1·27	1·04	0·79, 1·36	0·632	
60+	1·00	0·83	0·64, 1·08	0·97	0·74, 1·26	1·05	0·83, 1·32	0·458	
Sex									0·170
Men	1·00	0·90	0·72, 1·11	1·03	0·82, 1·28	1·07	0·86, 1·33	0·333	
Women	1·00	0·85	0·69, 1·05	0·95	0·77, 1·17	0·88	0·70, 1·11	0·422	
Race									0·033
NH White	1·00	0·86	0·71, 1·06	1·00	0·82, 1·21	1·03	0·83, 1·27	0·585	
NH Black	1·00	1·00	0·72, 1·38	1·12	0·83, 1·52	1·22	0·89, 1·66	0·134	
Mexican American	1·00	0·98	0·70, 1·39	1·00	0·71, 1·42	0·94	0·70, 1·28	0·749	
Other race/ethnic	1·00	0·82	0·56, 1·22	0·77	0·53, 1·13	0·60	0·42, 0·86	0·009	
Education									0·032
< High school	1·00	1·02	0·76, 1·39	1·28	0·95, 1·72	1·08	0·77, 1·51	0·355	
High school	1·00	1·00	0·75, 1·34	1·09	0·80, 1·48	1·54	1·08, 2·20	0·019	
Some college	1·00	0·78	0·60, 1·01	0·83	0·65, 1·06	0·77	0·59, 1·01	0·082	
College and above	1·00	0·86	0·59, 1·28	1·01	0·74, 1·37	0·90	0·64, 1·25	0·714	
Smoking									0·832
Never	1·00	0·94	0·77, 1·15	1·09	0·91, 1·30	0·99	0·81, 1·22	0·705	
Former	1·00	0·79	0·60, 1·05	0·85	0·64, 1·14	0·92	0·69, 1·23	0·682	
Current smoker	1·00	0·88	0·63, 1·25	0·96	0·67, 1·36	0·98	0·66, 1·45	0·951	
Alcohol drinking (past 12 months)									0·782
No	1·00	0·92	0·66, 1·29	0·90	0·63, 1·27	1·28	0·87, 1·87	0·279	
Yes	1·00	0·86	0·68, 1·09	1·07	0·86, 1·32	0·89	0·71, 1·13	0·696	
Missing	1·00	0·93	0·68, 1·29	0·79	0·56, 1·12	1·02	0·71, 1·46	0·790	
Leisure-time physical activity									0·291
< 600 MET-min/week	1·00	0·87	0·66, 1·14	1·17	0·91, 1·51	1·07	0·83, 1·39	0·215	
600–1200 MET-min/week	1·00	1·30	0·80, 2·10	1·15	0·70, 1·89	0·81	0·49, 1·33	0·321	
> 1200 MET-min/week	1·00	0·82	0·66, 1·02	0·82	0·65, 1·05	0·95	0·76, 1·20	0·665	
Ratio of family income:poverty									0·641
< 1·30	1·00	0·82	0·63, 1·06	1·07	0·80, 1·42	1·06	0·82, 1·37	0·403	
1·3–3·5	1·00	0·96	0·76, 1·22	0·96	0·74, 1·24	0·97	0·77, 1·23	0·813	
> 3·5	1·00	0·83	0·61, 1·12	1·06	0·84, 1·36	0·95	0·71, 1·25	0·866	
Dietary supplement use									0·444
No	1·00	0·89	0·72, 1·10	0·92	0·74, 1·14	1·10	0·89, 1·36	0·463	
Yes	1·00	0·86	0·70, 1·07	1·01	0·81, 1·26	0·88	0·70, 1·10	0·479	

NH, non-Hispanic.

Model adjusted for race, physical activity, education, income, smoking and alcohol drinking.

## Discussion

Using large, nationally representative samples from NHANES between 2005 and 2018, we estimated that the weighted prevalence of CircS was 37·3 %, indicating nearly four out of ten adults in the USA were affected, and observed no significant association between *n*-3 intake and CircS in the total study population. While the validity of our dietary data is supported by external consistency, a low level of *n*-3 intake and methodological limitations (errors in intake assessment and dietary misclassification) may together explain this null result.

The average daily intake of total *n*-3 FA in this study was 108·6 mg/d. This aligns with findings from other studies analysing NHANES data across overlapping timeframes, with minor differences falling within the normal variation range of population-based dietary surveys^(50–52)^. Specifically, the quartile distribution of total *n*-3 intake in this study is highly consistent with that reported in a recent study covering the same research period^(53)^. Furthermore, our study and latest NHANES-derived analyses jointly confirm that the average intake of *n*-3 in the US population remained far below recommended amounts^(54)^. This low exposure may explain the null association, as insufficient intake and the limited variation could limit the detection of meaningful effects on complex outcomes like CircS.

In fact, although our analyses yielded a null association, it does not necessarily negate the existence of a biological link between the two but rather raises two plausible interpretations. First, as discussed above, this null finding may stem from the dose and subcomponent constraints of *n*-3 intake in the NHANES dataset. Emerging evidence suggests that the efficacy of *n*-3’s anti-inflammatory effects on distinct health outcomes depends on both total intake and the ratio of EPA:DHA, with different outcomes requiring specific threshold doses^(55,56)^. Given that *n*-3 intake levels in the NHANES cohort are typically modest, it is plausible that the observed null association accurately reflects the inability of such intake levels to impact CircS – rather than the absence of a biological relationship. Second, the null association may be genuinely reflective of *n*-3’s mechanism-specific effects. While *n*-3 exerts well-documented anti-inflammatory effects, these effects may exhibit differential efficacy across health outcomes. Specifically, its anti-inflammatory properties appear to be more strongly associated with a lower prevalence of depression – possibly by targeting neuroinflammation in brain regions (e.g. the hippocampus) relevant to mood regulation^(55)^ yet insufficient to modulate the multi-factorial pathogenesis of CircS. This interpretation underscores the need for future studies to explore dose–response relationships and the differential effects of *n*-3 subcomponents (EPA *v*. DHA) on CircS, to clarify the conditions under which *n*-3 may exert regulatory effects on circadian health.

Additional potential explanations for this null result include intake assessment errors and dietary misclassification. First, self-reported dietary data from 24-h recalls and FFQ are prone to systematic bias. For example, pregnant women have been shown to overreport seafood intake relative to measured values, and fluctuations in non-daily foods (e.g. weekly fish consumption) further increase estimation errors, both contributing to widespread *n*-3 intake underestimation^(50,57,58)^. Second, the 24-h recall used in this study broadly categorises *n*-3-rich foods (e.g. grouping high-DHA deep-sea fish such as salmon with low-DHA freshwater fish such as carp). This causes imprecise intake estimates (under- or overreporting) that fail to reflect true *n*-3 exposure, ultimately weakening the potential association between *n*-3 intake and CircS^(59)^.

Notably, although no overall association was observed between dietary *n*-3 intake and CircS, our analysis revealed a component-specific positive association with depressive symptoms-a pattern consistent with emerging evidence. Researchers found that *n*-3 supplementation did not alter circadian activity rhythms or sleep structure but reduced depressive-like behaviours via lowering hippocampal IL-6 and elevating Brain-Derived Neurotrophic Factor^(60)^. Findings from another study showed EPA may affect Suprachiasmatic Nucleus (SCN) clock gene expression or melatonin secretion yet improved depression by promoting neurogenesis^(61)^. These studies suggest that *n*-3’s effects on depression-related behaviours may be independent of circadian regulation. Epidemiological evidence further validates this pattern. A 5-year prospective cohort study in Japan involving 44 611 participants revealed that dietary fish or *n*-3 intake was associated with a lower risk of depression^(62)^. This component-specific pattern is further corroborated by a meta-analysis including ten randomised controlled trials. It showed that only formulations with EPA accounting for over 60 % significantly reduce depressive symptoms, and their effects rely on NF-κB inhibition and neurogenesis rather than circadian rhythm pathways^(63)^. These collectively underscore that aggregate analyses of complex outcomes like CircS and *n*-3 intake may mask meaningful component-specific effects, especially when dimensions are governed by distinct biological pathways.

The results from subgroup analysis revealed that the association between *n*-3 and CircS varied across racial groups. Specifically, higher doses of *n*-3 intake were associated with a greater risk of CircS among NH Black and with lower odds of CircS among other races. It may be speculated that participants from different races have unique clock genes regulating the inflammatory and neurological pathways or the expression mechanisms of their clock genes differ from each other. In fact, a multitude of studies have indicated that people from diverse ethnic backgrounds possess unique genetic variations, which affect how their bodies process specific nutrients^(64,65)^, and sometimes nutrients such as fish oil supplementation were more like a moderator of their associations rather than a determining variable^(66)^. However, the exact mechanisms are not yet fully understood. The rationale could lie in the unique dietary patterns and habits these two races have^(67)^. *n*-3 Intake may lead to higher CircS risk if the diet contains large amounts of food with high fat^(68)^. A diet with unbalanced nutrition could also moderate the association between *n*-3 intake and CircS as the *ω*-6:*ω*-3 ratio may fluctuate and affect melatonin synthesis^(8)^. Moreover, given that the relationships between molecular clock alterations and inflammatory signalling were time-dependent^(69)^, the disrupted circadian rhythms resulting from specific lifestyles such as intensive activities at night could also explain some of the associations observed. Additional factors related to circadian rhythms include living environment, gut microbiota, physical activities and temperature^(70)^. However, all subgroup findings are exploratory in nature rather than confirmatory conclusions. Some subgroups may suffer from an insufficient sample size, which could lead to reduced statistical power and limit the robustness of their results.

This study has several strengths. First, this is the first study to investigate the association between *n*-3 intake and CircS. Although the primary finding of this study was a null result, it still validated the heterogeneous effect of *n*-3 on circadian- and depression-related outcomes that is easily masked by aggregate analyses. Notably, this study ensured the validity of outcome assessment through detailed, multi-dimensional measurement strategies. CircS was evaluated via a composite index of objective indicators and validated scales, while *n*-3 intake was quantified using a region-specific, validated FFQ. Second, the national sample used for analyses is representative and enables the generalisation of our findings to the broader US population. In addition, the findings of this study further suggest that future CircS-related studies should prioritise the adoption of a component-specific analysis strategy.

This study also has several limitations. First, the cross-sectional design fundamentally limits our ability to establish causal relationships between dietary *n*-3 intake and outcomes (CircS and depressive symptoms). Specifically, for the observed inverse association between EPA and depressive symptoms, we cannot rule out reverse causality bias: depressive symptoms may themselves alter dietary intake patterns and food quality^(71,72)^ – for example, reduced appetite, decreased consumption of *n*-3-rich foods (e.g. fish), or a preference for low-nutrient-density diets – thereby leading to lower dietary EPA intake. Although we adjusted for covariates related to diet quality (e.g. HEI) and general dietary supplement use to mitigate confounding, these adjustments cannot fully eliminate the impact of reverse causality, as depression-related dietary changes may involve unmeasured factors (e.g. emotional eating behaviours not captured by the HEI).

Second, potential exposure misclassification cannot be completely ruled out, primarily due to the use of self-reported data for dietary assessment using a 24-h food recall. Self-reported dietary data is inherently prone to recall bias (e.g. underreporting of high-fat foods or overestimation of ‘healthy’ foods). Such misclassification may weaken the potential association between *n*-3 intake and CircS, ultimately increasing the risk of false null findings. Although we adjusted for the general use of any dietary supplement as a covariate in our model, the inability to precisely quantify supplemental *n*-3 intake remains a limitation. This might have led to an underestimation of the total *n*-3 exposure for supplement users and could potentially dilute the observed associations.

Third, potential selection bias may exist in this study, reflected in baseline differences between included and excluded eligible participants (online Supplementary Table S3). This limits the generalisability of our findings to the full eligible population.

Additionally, while the study uses nationally representative data from NHANES 2005–2018, yielding good external validity for the non-institutionalised civilian population in the USA, the results may not be generalisable to other countries or regions – this is due to differences in dietary patterns (e.g. *n*-3 sources and intake levels), genetic backgrounds and environmental factors across populations.

Furthermore, regarding the positive findings for the depressive component, although it can be hypothesised that *n*-3 exerts its effects by inhibiting pro-inflammatory cytokines and promoting neurogenesis^(70)^, this mechanism is only applicable to the depression sub-phenotype and cannot be generalised to CircS as a whole. Residual confounding also remains a concern: despite adjusting for conventional confounders (age, gender, BMI), unmeasured variables such as gut microbiota characteristics^(73)^may still contribute to residual confounding.

### Conclusion

No associations between *n*-3 intake and CircS as a whole were observed in this cross-sectional study, but a higher *n*-3 intake was associated with lower odds of depressive symptoms. *n*-3 FA was identified as a risk factor among NH Black while a protective factor in other racial groups. Given the inconsistent findings regarding the benefits of *n*-3 in previous studies, general recommendations to increase its intake through fish-rich diets and supplements should be reconsidered. Further research is needed to explore the mechanisms underlying these findings.

## Supporting information

Zhang et al. supplementary material 1 Zhang et al. supplementary material

Zhang et al. supplementary material 2 Zhang et al. supplementary material

## Supplementary material

For supplementary material/s referred to in this article, please visit https://doi.org/10.1017/S0007114526106679.

## Table 1 Long description

A table comparing baseline characteristics of the study population by quartiles of n-3 fatty acid intake. The table has 30 rows and 14 columns. Column headers include Q1, Q2, Q3, Q4, and Total, each with Mean and SD sub-columns. Row labels include n, Age (years), Sex, Race, Education, Alcohol drinking (past 12 months), Smoking, Energy intake (kcal/d), n-3 FA intake (mg/d), EPA intake (mg/d), DHA intake (mg/d), Healthy Eating Index, Dietary supplement use, BMI (kg/m^2), Leisure-time physical activity, Ratio of family income:poverty, Central obesity, Elevated glucose, Elevated TAG, Reduced HDL-cholesterol, Elevated blood pressure, Depression, Short sleep, Metabolic syndrome, Circadian syndrome. Row 1: n, 3046, 2992, 2987, 3003, 12028. Row 2: Age (years), 480, 477, 475, 486, 480. Row 3: Sex, Men, 1314, 1393, 1583, 1598, 5888. Row 4: Sex, Women, 1732, 1599, 1404, 1405, 6140. Row 5: Race, NH White, 1588, 1483, 1314, 1149, 5534. Row 6: Race, NH Black, 470, 522, 638, 722, 2352. Row 7: Race, Mexican American, 391, 453, 515, 447, 1806. Row 8: Race, Other race/ethnic, 597, 534, 520, 685, 2336. Row 9: Education, < High school, 761, 657, 713, 625, 2756. Row 10: Education, High school, 731, 728, 677, 623, 2759. Row 11: Education, Some college, 902, 914, 886, 886, 3588. Row 12: Education, College and above, 650, 690, 709, 868, 2917. Row 13: Alcohol drinking (past 12 months), No, 512, 514, 479, 433, 1938. Row 14: Alcohol drinking (past 12 months), Yes, 1679, 1773, 1788, 1903, 7143. Row 15: Alcohol drinking (past 12 months), Missing, 855, 705, 720, 667, 2947. Row 16: Smoking, Never, 1622, 1613, 1619, 1700, 6554. Row 17: Smoking, Former, 761, 768, 784, 781, 3094. Row 18: Smoking, Current smoker, 663, 609, 584, 520, 2376. Row 19: Energy intake (kcal/d), 18185, 20850, 22387, 22724, 20971. Row 20: n-3 FA intake (mg/d), 72, 245, 579, 3660, 1096. Row 21: EPA intake (mg/d), 36, 78, 131, 727, 366. Row 22: DHA intake (mg/d), 36, 78, 131, 727, 366. Row 23: Healthy Eating Index, 443, 448, 456, 460, 451. Row 24: Dietary supplement use, 1549, 1554, 1550, 1715, 6368. Row 25: BMI (kg/m^2), 287, 290, 293, 291, 290. Row 26: Leisure-time physical activity, < 600 MET-min/week, 1309, 1170, 1162, 1059, 4700. Row 27: Leisure-time physical activity, 600-1200 MET-min/week, 311, 364, 339, 349, 1363. Row 28: Leisure-time physical activity, > 1200 MET-min/week, 1425, 1458, 1486, 1595, 5964. Row 29: Ratio of family income:poverty, < 130, 957, 866, 784, 660, 3267. Row 30: Ratio of family income:poverty, 130-35, 1073, 1099, 1082, 1060, 4314. Row 31: Ratio of family income:poverty, > 35, 785, 803, 886, 1030, 3504.

Navigate back to Table 1.

## Table 2 Long description

The table presents the association between dietary n-3 fatty acid (FA) intake and circadian syndrome among US adults, based on data from the National Health and Nutrition Examination Survey 2005-2018. The table is divided into three main sections: n-3 FA, EPA, and DHA, each with four models. Each section compares the odds ratios (OR) and 95% confidence intervals (CI) across quartiles of intake (Q1, Q2, Q3, Q4). The table includes the following columns: Q1, Q2 OR, Q2 95% CI, Q3 OR, Q3 95% CI, Q4 OR, Q4 95% CI, and P for trend. Row-wise data: n-3 FA Model 1: Q1 1.00, Q2 0.86, Q2 95% CI 0.74-0.99, Q3 0.96, Q3 95% CI 0.83-1.10, Q4 0.88, Q4 95% CI 0.75-1.04, P for trend 0.300. n-3 FA Model 2: Q1 1.00, Q2 0.88, Q2 95% CI 0.76-1.02, Q3 1.02, Q3 95% CI 0.88-1.18, Q4 0.98, Q4 95% CI 0.83-1.15, P for trend 0.779. n-3 FA Model 3: Q1 1.00, Q2 0.89, Q2 95% CI 0.76-1.03, Q3 1.04, Q3 95% CI 0.90-1.20, Q4 1.03, Q4 95% CI 0.87-1.22, P for trend 0.360. n-3 FA Model 4: Q1 1.00, Q2 0.89, Q2 95% CI 0.76-1.03, Q3 1.03, Q3 95% CI 0.90-1.19, Q4 1.03, Q4 95% CI 0.87-1.22, P for trend 0.359. EPA Model 1: Q1 1.00, Q2 1.00, Q2 95% CI 0.84-1.20, Q3 1.03, Q3 95% CI 0.88-1.21, Q4 0.89, Q4 95% CI 0.76-1.06, P for trend 0.221. EPA Model 2: Q1 1.00, Q2 1.02, Q2 95% CI 0.86-1.21, Q3 1.06, Q3 95% CI 0.90-1.26, Q4 0.99, Q4 95% CI 0.83-1.17, P for trend 0.974. EPA Model 3: Q1 1.00, Q2 1.01, Q2 95% CI 0.85-1.21, Q3 1.05, Q3 95% CI 0.89-1.25, Q4 1.02, Q4 95% CI 0.86-1.21, P for trend 0.703. EPA Model 4: Q1 1.00, Q2 1.01, Q2 95% CI 0.85-1.20, Q3 1.06, Q3 95% CI 0.89-1.25, Q4 1.02, Q4 95% CI 0.86-1.21, P for trend 0.668. DHA Model 1: Q1 1.00, Q2 0.84, Q2 95% CI 0.72-0.98, Q3 0.92, Q3 95% CI 0.79-1.06, Q4 0.87, Q4 95% CI 0.74-1.03, P for trend 0.180. DHA Model 2: Q1 1.00, Q2 0.88, Q2 95% CI 0.76-1.03, Q3 0.98, Q3 95% CI 0.84-1.13, Q4 0.96, Q4 95% CI 0.82-1.14, P for trend 0.918. DHA Model 3: Q1 1.00, Q2 0.89, Q2 95% CI 0.77-1.04, Q3 1.00, Q3 95% CI 0.86-1.16, Q4 1.02, Q4 95% CI 0.86-1.20, P for trend 0.573. DHA Model 4: Q1 1.00, Q2 0.89, Q2 95% CI 0.76-1.04, Q3 0.99, Q3 95% CI 0.86-1.15, Q4 1.02, Q4 95% CI 0.86-1.21, P for trend 0.576.

Navigate back to Table 2.

## Table 3 Long description

A table comparing the odds ratios (OR) and 95 percent confidence intervals (CI) for components of circadian syndrome across quartiles of n-3 fatty acid (FA) intake. The table has 21 rows and 10 columns. The columns are labeled as Q1, Q2 OR, Q2 95 % CI, Q3 OR, Q3 95 % CI, Q4 OR, Q4 95 % CI, and P for trend. The rows are grouped into three sections: n-3 FA, EPA, and DHA. Each section lists different components: Central obesity, Elevated glucose, Elevated TAG, Low HDL-cholesterol, Elevated blood pressure, Depressive symptom, and Short sleep. Each row provides the OR and 95 % CI for Q2, Q3, and Q4 compared to Q1, along with the P for trend value. Notable trends include a significant decrease in the risk of depressive symptoms with increasing n-3 FA, EPA, and DHA intake, as indicated by the P for trend values less than 0.001.

Navigate back to Table 3.

## Table 4 Long description

A table comparing the association between quartiles of n-3 fatty acid intake and circadian syndrome across different subgroups. The table has 12 rows and 13 columns. The columns are labeled as Q1, Q2 OR, Q2 95 % CI, Q3 OR, Q3 95 % CI, Q4 OR, Q4 95 % CI, P for trend, and P for interaction. The rows are labeled as Age, Sex, Race, Education, Smoking, Alcohol drinking (past 12 months), Leisure-time physical activity, and Ratio of family income:poverty. Each row provides odds ratios (OR) and 95 percent confidence intervals (CI) for different quartiles of n-3 fatty acid intake. The table also includes P values for trend and interaction across the subgroups. Row 1: Age 20-39, Q1 1.00, Q2 0.94, Q2 95 % CI 0.69-1.28, Q3 0.95, Q3 95 % CI 0.69-1.33, Q4 0.67, Q4 95 % CI 0.48-0.95, P for trend 0.054, P for interaction 0.317. Row 2: Age 40-59, Q1 1.00, Q2 0.86, Q2 95 % CI 0.67-1.11, Q3 1.00, Q3 95 % CI 0.78-1.28, Q4 1.03, Q4 95 % CI 0.78-1.37, P for trend 0.603, P for interaction 0.317. Row 3: Age 60+, Q1 1.00, Q2 0.85, Q2 95 % CI 0.64-1.14, Q3 1.03, Q3 95 % CI 0.80-1.32, Q4 1.05, Q4 95 % CI 0.83-1.33, P for trend 0.342, P for interaction 0.317. Row 4: Sex Men, Q1 1.00, Q2 0.87, Q2 95 % CI 0.72-1.04, Q3 1.15, Q3 95 % CI 0.95-1.39, Q4 1.08, Q4 95 % CI 0.87-1.33, P for interaction 0.664. Row 5: Sex Women, Q1 1.00, Q2 0.91, Q2 95 % CI 0.72-1.14, Q3 0.93, Q3 95 % CI 0.75-1.16, Q4 0.92, Q4 95 % CI 0.74-1.16, P for interaction 0.664. Row 6: Race NH White, Q1 1.00, Q2 0.87, Q2 95 % CI 0.72-1.06, Q3 1.07, Q3 95 % CI 0.89-1.30, Q4 1.05, Q4 95 % CI 0.85-1.30, P for interaction 0.008. Row 7: Race NH Black, Q1 1.00, Q2 1.10, Q2 95 % CI 0.81-1.50, Q3 1.16, Q3 95 % CI 0.87-1.54, Q4 1.36, Q4 95 % CI 1.02-1.82, P for interaction 0.008. Row 8: Race Mexican American, Q1 1.00, Q2 0.78, Q2 95 % CI 0.53-1.14, Q3 0.87, Q3 95 % CI 0.59-1.29, Q4 0.83, Q4 95 % CI 0.60-1.14, P for interaction 0.008. Row 9: Race Other race/ethnic, Q1 1.00, Q2 0.85, Q2 95 % CI 0.59-1.23, Q3 0.78, Q3 95 % CI 0.52-1.17, Q4 0.57, Q4 95 % CI 0.39-0.85, P for interaction 0.008. Row 10: Education < High school, Q1 1.00, Q2 1.17, Q2 95 % CI 0.86-1.58, Q3 1.22, Q3 95 % CI 0.90-1.64, Q4 1.11, Q4 95 % CI 0.80-1.55, P for interaction 0.105. Row 11: Education High school, Q1 1.00, Q2 0.84, Q2 95 % CI 0.63-1.12, Q3 1.16, Q3 95 % CI 0.84-1.61, Q4 1.38, Q4 95 % CI 0.93-2.04, P for interaction 0.105. Row 12: Education Some college, Q1 1.00, Q2 0.78, Q2 95 % CI 0.61-1.01, Q3 0.89, Q3 95 % CI 0.69-1.16, Q4 0.79, Q4 95 % CI 0.60-1.04, P for interaction 0.105. Row 13: Education College and above, Q1 1.00, Q2 0.96, Q2 95 % CI 0.65-1.43, Q3 1.07, Q3 95 % CI 0.77-1.47, Q4 1.00, Q4 95 % CI 0.72-1.38, P for interaction 0.105.

Navigate back to Table 4.

## Table 5 Long description

A table comparing the association between quartiles of EPA intake and circadian syndrome across different subgroups. The table has 12 rows and 13 columns. The columns are labeled as follows: Q1, Q2 OR, Q2 95 % CI, Q3 OR, Q3 95 % CI, Q4 OR, Q4 95 % CI, P for trend, P for interaction. The rows are labeled as follows: Age, Sex, Race, Education, Smoking, Alcohol drinking (past 12 months), Leisure-time physical activity, Ratio of family income:poverty, Dietary supplement use. Each row provides odds ratios (OR) and 95 percent confidence intervals (CI) for different quartiles of EPA intake (Q1, Q2, Q3, Q4) and includes P values for trend and interaction. Row 1: Age 20-39, Age 40-59, Age 60+. Row 2: Sex Men, Sex Women. Row 3: Race NH White, Race NH Black, Race Mexican American, Race Other race/ethnic. Row 4: Education < High school, Education High school, Education Some college, Education College and above. Row 5: Smoking Never, Smoking Former, Smoking Current smoker. Row 6: Alcohol drinking (past 12 months) No, Alcohol drinking (past 12 months) Yes, Alcohol drinking (past 12 months) Missing. Row 7: Leisure-time physical activity < 600 MET-min/week, Leisure-time physical activity 600-1200 MET-min/week, Leisure-time physical activity > 1200 MET-min/week. Row 8: Ratio of family income:poverty < 1.30, Ratio of family income:poverty 1.3-3.5, Ratio of family income:poverty > 3.5. Row 9: Dietary supplement use No, Dietary supplement use Yes.

Navigate back to Table 5.

## Table 6 Long description

Navigate back to Table 6.
