# Supplementary material for: n-3 Fatty acid intake and circadian syndrome in US adults: evidence from the National Health and Nutrition Examination Survey 2005–2018
Source: Br J Nutr. 2026 Mar 3;135(12):1351–63. doi: 10.1017/S0007114526106679 (PMC13423522; doi:10.1017/S0007114526106679)
Supplement: Zhang et al. supplementary material 1 — Zhang et al. supplementary material [file S0007114526106679sup001.docx]

Supplimentary table

| Table S1. Baseline characteristics of the study population, stratified by quartiles of omega-3 fatty acid intake: NHANES 2005-2018 | | | | | | |
| --- | --- | --- | --- | --- | --- | --- |
|  | Quartiles of omega-3 FA | | | | | |
|  | Q1 | Q2 | Q3 | Q4 | Total | p-value |
| N | 2,960 (25.9%) | 2,955 (25.9%) | 2,935 (24.3%) | 2,949 (23.9%) | 11,799 (100.0%) |  |
| Age (years) | 48.1 (15.4) | 47.7 (15.3) | 47.8 (15.0) | 48.7 (14.7) | 48.1 (15.1) | 0.303 |
| Gender |  |  |  |  |  |  |
| Men | 1,272 (41.3%) | 1,365 (48.2%) | 1,531 (51.1%) | 1,552 (51.9%) | 5,720 (48.0%) | <0.001 |
| Women | 1,688 (58.7%) | 1,590 (51.8%) | 1,404 (48.9%) | 1,397 (48.1%) | 6,079 (52.0%) |  |
| Race |  |  |  |  |  |  |
| NH White | 1,539 (74.4%) | 1,464 (72.7%) | 1,293 (68.7%) | 1,132 (63.8%) | 5,428 (70.0%) | <0.001 |
| NH Black | 458 (8.2%) | 513 (9.0%) | 629 (11.6%) | 700 (12.6%) | 2,300 (10.3%) |  |
| Mex American | 380 (6.4%) | 448 (7.5%) | 499 (8.9%) | 437 (8.0%) | 1,764 (7.7%) |  |
| Other race/ethnic | 583 (10.9%) | 530 (10.9%) | 514 (10.8%) | 680 (15.6%) | 2,307 (12.0%) |  |
| Education |  |  |  |  |  |  |
| <High school | 743 (16.7%) | 645 (14.0%) | 693 (14.4%) | 614 (12.4%) | 2,695 (14.4%) | <0.001 |
| High school | 715 (24.9%) | 713 (24.6%) | 657 (21.6%) | 614 (20.9%) | 2,699 (23.1%) |  |
| Some college | 874 (31.8%) | 906 (31.5%) | 878 (31.9%) | 868 (31.2%) | 3,526 (31.6%) |  |
| college and above | 626 (26.6%) | 688 (29.8%) | 705 (32.1%) | 852 (35.5%) | 2,871 (30.9%) |  |
| Alcohol drinking (past 12 months) |  |  |  |  |  |  |
| No | 502 (13.7%) | 510 (14.0%) | 475 (12.2%) | 426 (11.8%) | 1,913 (13.0%) | 0.004 |
| Yes | 1,626 (59.5%) | 1,745 (62.5%) | 1,748 (65.3%) | 1,864 (65.9%) | 6,983 (63.2%) |  |
| Missing | 832 (26.8%) | 700 (23.5%) | 712 (22.4%) | 659 (22.3%) | 2,903 (23.8%) |  |
| Smoking |  |  |  |  |  |  |
| Never | 1,575 (53.0%) | 1,608 (55.2%) | 1,595 (55.0%) | 1,671 (55.8%) | 6,449 (54.7%) | 0.010 |
| Former | 744 (25.0%) | 757 (25.2%) | 768 (26.9%) | 774 (28.1%) | 3,043 (26.3%) |  |
| Current smoker | 641 (21.9%) | 588 (19.6%) | 572 (18.1%) | 502 (16.1%) | 2,303 (19.0%) |  |
| Energy intake (kcal/day) | 1792.4 (585.2) | 2049.3 (603.9) | 2178.9 (642.2) | 2190.0 (668.3) | 2047.7 (640.4) | <0.001 |
| Omega-3 FA intake (mg/d) | 7.0 (3.7) | 24.0 (5.7) | 56.9 (14.0) | 363.2 (383.6) | 108.6 (224.2) | <0.001 |
| EPA intake (mg/d) | 3.5 (2.5) | 7.7 (4.3) | 12.8 (7.6) | 127.1 (154.6) | 36.4 (86.9) | <0.001 |
| DHA intake (mg/d) | 3.5 (2.6) | 16.3 (6.7) | 44.1 (12.7) | 236.2 (237.9) | 72.2 (141.1) | <0.001 |
| Healthy eating index | 44.4 (11.8) | 44.8 (10.9) | 45.7 (10.8) | 49.8 (11.0) | 46.1 (11.3) | <0.001 |
| Dietary supplement use | 1,510 (53.4%) | 1,534 (53.6%) | 1,535 (54.3%) | 1,689 (60.3%) | 6,268 (55.3%) | <0.001 |
| BMI (kg/m2) | 28.7 (5.8) | 29.0 (5.9) | 29.3 (6.1) | 29.1 (6.4) | 29.0 (6.0) | 0.071 |
| Leisure time physical activity |  |  |  |  |  |  |
| <600 MET-min/week | 1,271 (38.4%) | 1,169 (34.6%) | 1,157 (34.0%) | 1,042 (31.1%) | 4,639 (34.6%) | 0.001 |
| 600-1200 MET-min/week | 305 (10.4%) | 356 (12.7%) | 332 (11.7%) | 350 (12.9%) | 1,343 (11.9%) |  |
| >1200 MET-min/week | 1,383 (51.3%) | 1,430 (52.7%) | 1,446 (54.3%) | 1,557 (56.1%) | 5,816 (53.5%) |  |
| Ratio of family income to poverty |  |  |  |  |  |  |
| <1.30 | 930 (24.1%) | 856 (20.8%) | 768 (17.7%) | 631 (15.1%) | 3,185 (19.5%) | <0.001 |
| 1.3-3.5 | 1,043 (36.9%) | 1,084 (38.9%) | 1,072 (37.3%) | 1,041 (34.7%) | 4,240 (37.0%) |  |
| >3.5 | 764 (39.0%) | 791 (40.3%) | 868 (45.0%) | 1,023 (50.2%) | 3,446 (43.5%) |  |
| Central obesity | 1,747 (59.6%) | 1,758 (57.6%) | 1,740 (58.5%) | 1,635 (55.4%) | 6,880 (57.8%) | 0.081 |
| Elevated glucose | 1,591 (49.4%) | 1,585 (51.0%) | 1,632 (52.6%) | 1,668 (52.8%) | 6,476 (51.4%) | 0.196 |
| Elevated triglycerides | 1,298 (41.6%) | 1,251 (39.4%) | 1,231 (38.9%) | 1,267 (40.9%) | 5,047 (40.2%) | 0.325 |
| Reduced HDL-C | 1,402 (44.5%) | 1,329 (42.1%) | 1,380 (44.1%) | 1,306 (42.5%) | 5,417 (43.3%) | 0.475 |
| Elevated blood pressure | 1,468 (42.8%) | 1,430 (43.3%) | 1,491 (45.9%) | 1,455 (42.8%) | 5,844 (43.7%) | 0.262 |
| Depression | 781 (25.2%) | 744 (22.7%) | 605 (18.4%) | 609 (18.6%) | 2,739 (21.3%) | <0.001 |
| Short sleep | 969 (30.1%) | 964 (29.2%) | 973 (28.6%) | 975 (28.7%) | 3,881 (29.2%) | 0.695 |
| Metabolic syndrome | 1,504 (46.5%) | 1,431 (44.3%) | 1,501 (47.4%) | 1,445 (45.6%) | 5,881 (45.9%) | 0.340 |
| Circadian syndrome | 1,273 (38.4%) | 1,209 (35.7%) | 1,252 (37.9%) | 1,218 (36.9%) | 4,952 (37.2%) | 0.332 |
| Data presented as weighted means and standard deviations for continuous measures and unweighted N (weighted %) for categorical measures. P-values based on linear regression for continuous measures and logistic regression for categorical measures. | | | | | | |

*Note: This sensitivity analysis applied a conservative cut-off for energy intake (3,500 kcal/day for women and 4,200 kcal/day for men).

Table S2. Weighted odds ratios (95% CI) for components of Circadian Syndrome across quartiles of omega-3 fatty acid intake among Black adults: NHANES 2005-2018

|  | Quartiles of intake | | | |  |
| --- | --- | --- | --- | --- | --- |
|  | Q1 | Q2 | Q3 | Q4 | p-value |
| omega-3 FA |  |  |  |  |  |
| Central obesity | 1.00 | 1.04 (0.77-1.42) | 1.20 (0.89-1.62) | 1.09 (0.83-1.43) | 0.421 |
| Elevated glucose | 1.00 | 1.08 (0.81-1.46) | 1.01 (0.74-1.38) | 1.13 (0.84-1.51) | 0.515 |
| Elevated triglyceride | 1.00 | 0.94 (0.67-1.31) | 0.99 (0.76-1.31) | 1.04 (0.77-1.40) | 0.659 |
| Low HDL | 1.00 | 0.87 (0.63-1.19) | 1.21 (0.94-1.56) | 1.09 (0.83-1.44) | 0.167 |
| Elevated blood pressure | 1.00 | 1.13 (0.83-1.54) | 1.12 (0.83-1.51) | 1.17 (0.86-1.58) | 0.365 |
| Depressive symptom | 1.00 | 0.97 (0.63-1.48) | 0.82 (0.58-1.16) | 0.85 (0.63-1.16) | 0.153 |
| Short sleep | 1.00 | 0.92 (0.69-1.24) | 1.08 (0.80-1.45) | 1.05 (0.78-1.42) | 0.512 |
| EPA |  |  |  |  |  |
| Central obesity | 1.00 | 1.01 (0.73-1.39) | 1.15 (0.86-1.55) | 1.25 (0.92-1.70) | 0.116 |
| Elevated glucose | 1.00 | 1.04 (0.79-1.37) | 0.98 (0.76-1.26) | 1.20 (0.93-1.55) | 0.224 |
| Elevated triglyceride | 1.00 | 1.01 (0.70-1.44) | 1.01 (0.76-1.34) | 1.02 (0.78-1.34) | 0.890 |
| Low HDL | 1.00 | 0.88 (0.63-1.23) | 1.03 (0.81-1.31) | 1.06 (0.81-1.38) | 0.439 |
| Elevated blood pressure | 1.00 | 1.06 (0.75-1.50) | 1.02 (0.75-1.38) | 1.22 (0.88-1.68) | 0.261 |
| Depressive symptom | 1.00 | 0.71 (0.50-1.01) | 0.82 (0.61-1.12) | 0.76 (0.54-1.06) | 0.170 |
| Short sleep | 1.00 | 1.05 (0.82-1.33) | 1.20 (0.90-1.60) | 1.09 (0.82-1.46) | 0.452 |
| DHA |  |  |  |  |  |
| Central obesity | 1.00 | 0.89 (0.66-1.21) | 1.10 (0.83-1.46) | 0.94 (0.71-1.24) | 0.999 |
| Elevated glucose | 1.00 | 1.09 (0.82-1.46) | 1.01 (0.73-1.38) | 1.10 (0.82-1.48) | 0.653 |
| Elevated triglyceride | 1.00 | 0.84 (0.59-1.19) | 0.99 (0.74-1.32) | 0.99 (0.72-1.36) | 0.704 |
| Low HDL | 1.00 | 0.89 (0.65-1.22) | 1.17 (0.88-1.55) | 1.03 (0.79-1.36) | 0.376 |
| Elevated blood pressure | 1.00 | 1.05 (0.76-1.46) | 1.07 (0.83-1.37) | 1.14 (0.87-1.51) | 0.328 |
| Depressive symptom | 1.00 | 0.75 (0.53-1.08) | 0.80 (0.58-1.09) | 0.71 (0.53-0.96) | 0.040 |
| Short sleep | 1.00 | 0.90 (0.68-1.17) | 1.10 (0.83-1.46) | 1.02 (0.75-1.39) | 0.579 |
| Model adjusted for age, gender, energy intake, race, physical activity, education, income, smoking, and alcohol drinking. | | | | | |

| Table S3. Comparison of characteristics between included and excluded participants:  NHANES 2005-2018 | | | | |
| --- | --- | --- | --- | --- |
|  | Inclusion of participants | | | |
|  | Excluded | Included | Total | p-value |
| N | 27,721 (69.7%) | 12,028 (30.3%) | 39,749 (100.0%) |  |
| Age (years) | 49.4 (18.2) | 50.3 (17.6) | 49.7 (18.0) | <0.001 |
| Gender |  |  |  |  |
| Men | 13,362 (48.2%) | 5,888 (49.0%) | 19,250 (48.4%) | 0.169 |
| Women | 14,359 (51.8%) | 6,140 (51.0%) | 20,499 (51.6%) |  |
| Race |  |  |  |  |
| NH White | 11,009 (39.7%) | 5,534 (46.0%) | 16,543 (41.6%) | <0.001 |
| NH Black | 6,248 (22.5%) | 2,352 (19.6%) | 8,600 (21.6%) |  |
| Mex American | 4,407 (15.9%) | 1,806 (15.0%) | 6,213 (15.6%) |  |
| Other race/ethn | 6,057 (21.8%) | 2,336 (19.4%) | 8,393 (21.1%) |  |
| Education |  |  |  |  |
| <High school | 7,334 (26.5%) | 2,756 (22.9%) | 10,090 (25.4%) | <0.001 |
| High school | 6,343 (22.9%) | 2,759 (23.0%) | 9,102 (22.9%) |  |
| Some college | 7,973 (28.8%) | 3,588 (29.9%) | 11,561 (29.1%) |  |
| college and above | 6,018 (21.8%) | 2,917 (24.3%) | 8,935 (22.5%) |  |
| Alcohol drinking (past 12 months) |  |  |  |  |
| No | 3,627 (13.1%) | 1,938 (16.1%) | 5,565 (14.0%) | <0.001 |
| Yes | 12,494 (45.1%) | 7,143 (59.4%) | 19,637 (49.4%) |  |
| Missing | 11,600 (41.8%) | 2,947 (24.5%) | 14,547 (36.6%) |  |
| Smoking |  |  |  |  |
| Never | 15,526 (56.1%) | 6,554 (54.5%) | 22,080 (55.6%) | <0.001 |
| Former | 6,413 (23.2%) | 3,094 (25.7%) | 9,507 (23.9%) |  |
| Current smoker | 5,754 (20.8%) | 2,376 (19.8%) | 8,130 (20.5%) |  |
